# Criteria-based ranking (CBR): A comprehensive process for selecting and prioritizing monitoring indicators

**DOI:** 10.1016/j.mex.2018.10.015

**Published:** 2018-10-23

**Authors:** Elaine Ho

**Affiliations:** School of Environment, Resources and Sustainability, Canadian Water Network and Canadian Rivers Institute at the University of Waterloo, 200 University Avenue West, Waterloo, Ontario, N2L 3G1, Canada

**Keywords:** Criteria-based ranking (CBR) process (for indicator selection/prioritization), Valued ecosystem components (VECs), Indicators, Monitoring, Watershed monitoring, Water monitoring, Indicator selection process

## Abstract

Resources allocated to natural resource management often fluctuate, requiring the types and numbers of parameters used in monitoring programs (e.g., indicators of ecosystem health) to be frequently reassessed. Conventional approaches to selecting monitoring indicators are often biased and non-inclusive. A new Criteria-based Ranking (CBR) process for selecting and/or prioritizing indicators was tested in the Muskoka River Watershed (Ontario, Canada). The CBR process is based on two environmental assessment tools, Simple Weighted and Leopold matrices. It incorporates environmental components and criteria for assessing each indicator, which generate a score per indicator. The process tested in this study was concluded to be an effective way to prioritize and/or select environmental monitoring indicators. A different set of indicators emerged when a common set of criteria was used to assess monitoring indicators. Benefits of the CBR process include:

• Standardization of indicator selection process with less bias and lower cost (e.g., time and human resources).
• Indicators that are representative of the community and more relevant for decision-making (e.g., more resilient to socio-political change).
• Adaptability: (1) to other goals, e.g., selecting from a list of Valued Ecosystem Components (VECs), and (2) to any context through localized scoring criteria. Easily integrated into existing practice.

## Graphical abstract

**Figure:**
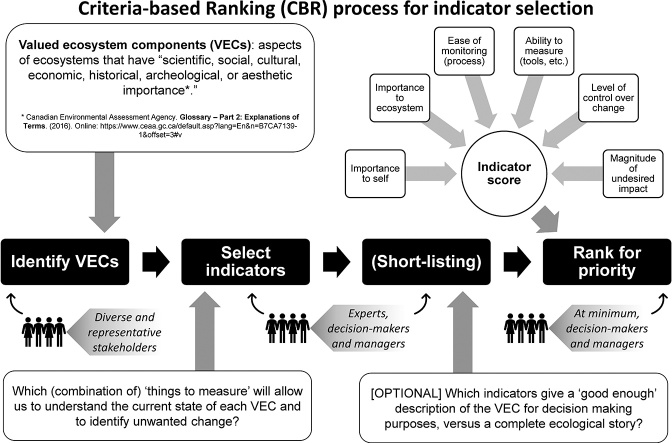

**Specifications Table**

**Table:** 

Subject Area	Environmental Science
More specific subject area:	Water monitoring
Method name:	Criteria-based ranking (CBR) process (for indicator selection/prioritization)
Name and reference of original method	N/A
Resource availability	N/A

## Method details

### Introduction

Resources allocated to natural resource management often fluctuates. As a result, the types and numbers of parameters (e.g., indicators for ecosystem health) being measured in monitoring programs are frequently reassessed according to management (or political) priorities and limits on budgets and human resources [1]. This periodic need to refocus monitoring conflicts with the need to maintain consistent, long-term indicators which demonstrate changes to ecosystem health or define ‘abnormal’ indicator measures.

To mitigate the conflict between updating monitoring indicators according to current needs or limitations and maintaining long-term indicators, a new process for selecting and prioritizing indicators is needed. This new process should make shortlisting indicators possible and relevant while ensuring continuity of long-term indicators. Further, the process of selecting indicators should be robust and comprehensive enough to represent broad perspectives representative of diverse decision-makers, technical experts (e.g., scientists) and local stakeholders to improve resilience against changing political regimes.

Conventional processes for selecting or short-listing environmental monitoring indicators generally involve two steps: identify Valued Ecosystem Components (VECs) – things people care about (e.g., swimmable waters, fishing opportunities) – and discussing which indicators and methods should be used to measure them. VECs create a scope for the selection of indicators. Discussions on indicator selection are highly-dependent on who is present in the room, especially when the monitoring program is long-standing with a consistent group of discussants from year to year. A new process for selecting or prioritizing monitoring indicators was synthesized in the context of watershed management from existing approaches in environmental assessment, rooted in management studies.

### The criteria-based ranking (CBR) process

In management studies, Multiple Criteria Decision Making (MCDM) is the closest approach to the new method presented here. MCDM was first formally discussed in the 1970s by management scientist Dr. Stanley Zionts and colleagues [2]. Generally, MDCM methods outline the following three steps [3]: determine relevant criteria and alternatives, attach numerical measures to relative importance of criteria and impact of alternatives, and process numerical values to determine a rank.

The Criteria-Based Ranking (CBR) process presented in this paper borrowed design aspects from Simple Weighted and Leopold matrices used in environmental assessment [4,5]. Both matrices score criteria that together calculate a rank. A Simple Weighted matrix places environmental components down the left column, project actions (stressors) along the top row, and a score of impact in each intersecting box (positive or negative, from no impact to severe/permanent impact). In Simple Weighted matrices of environmental assessment, ecosystem components are weighted so each score is multiplied by the weight and summed up at the end of each row [5]; weighting was not applied in this study.

Since the CBR process aims to prioritize indicators rather than quantify stressors and impacts, the process used in this research used a modified table (Table 1). The process includes environmental components along the left column, and criteria for assessing each indicator along the top row. Like in Simple Weighted matrices, a single score is placed in each intersecting box, which is summed at the end of each row (the indicator’s total score). Criteria incorporate basic principles of Leopold matrices – importance (of the component) and magnitude of impact – in addition to other principles relevant to the watershed management context (e.g., ease of monitoring).

**Table 1:** Sample prioritization matrix adapted from environmental assessment tools.

Criteria (score: 1–5)	Indicator		
	Secchi depth	Algae biomass	Calcium
Cost-effective	4	2	3
Ease of measuring	5	1	4
Important to me	2	4	3

**Total Score**	**11**	**7**	**10**

The monitoring program in the Muskoka River Watershed (Ontario, Canada) was used as a case study to assess whether using the CBR process would have any effect on the process and outcome of short-listing monitoring indicators. This study was narrowly scoped since engaging with stakeholders for assessing VECs, a long list of indicators and criteria for short-listing would have required large amounts of resources (time, human resources, funds) to implement. As such, the first question was to test the final CBR process to assess whether spending resources to implement it full-scale would provide value. A workshop was held with Muskoka Watershed Council on August 5, 2018 to test the CBR process and answer this question.

Workshop participants were tasked with reducing a list of six indicators to a list of five using the CBR process. The six indicators used were Secchi depth, algae growth, calcium, land use, wetland cover and carbon footprint. SurveyMonkey was used to collect scores for each indicator from each workshop participant, which was then summed up into the matrix exemplified in Table 1. SurveyMonkey was used for ease of response by participants, as well as to ensure participants could not easily track scores across criteria to manipulate scores to individual interests. Each question on the survey was dedicated to each indicator. The following is Question 2 of the survey, with the seven criteria that were used to assess all indicators:

• *Rank the indicator 'secchi depth' on a scale of 1 (least) to 5 (most) based on the criteria below.*
  ○ *I would include this indicator, by this or other name, in the Report Card (e.g. not just in the Background Report)*
  ○ *This indicator is measurable given reasonably expected resources (tools, people, funds, time…)*
  ○ *We have control over changes to this indicator*
  ○ *We have effective mechanisms for correcting CURRENT unwanted changes to this indicator*
  ○ *We have effective mechanisms for correcting FUTURE unwanted changes to this indicator*
  ○ *Unwanted changes to this indicator would result in serious impacts (directly or indirectly) on ecological and human systems*
  ○ *This indicator is important to me*

**Fig. 1:**
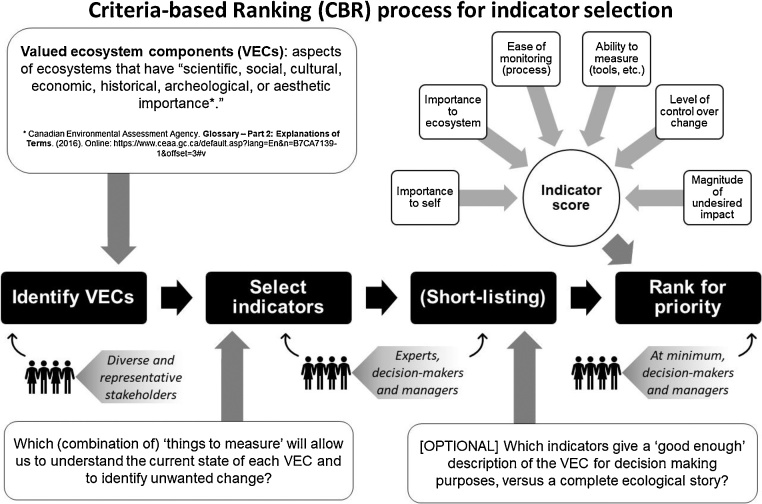
Criteria-based ranking (CBR) process for indicator selection, highlighted in the box. Criteria used in the figure are examples of what may be considered and can be tailored to specific contexts.

The process used for this first iteration of the CBR method is outlined in Fig. 1. The first two steps are typical of conventional approaches, except that broader stakeholder engagement is strongly encouraged in the CBR process.

### Results

The question going into the workshop was whether the CBR process would be an effective way to assess monitoring indicators moving forward. Table 2 summarizes the total scores from all participants for each criterion and indicator.

**Table 2:** Results of the indicator prioritization exercise. Scores are the sum of individual participants’ scores for each criterion.

Criteria	Indicator*					
	Secchi Depth	Algae	Calcium	Land Use	Wetland cover	Footprint (new)
I would include this indicator, by this or other name, in the Report Card (e.g. not just in the Background Report)	17	31	23	33	32	27
This indicator is measurable given reasonably expected resources (tools, people, funds, time…)	33	22	25	30	25	20
We have control over changes to this indicator	18	20	18	27	24	23
We have effective mechanisms for correcting CURRENT unwanted changes to this indicator	16	19	16	25	19	20
We have effective mechanisms for correcting FUTURE unwanted changes to this indicator	20	21	17	27	21	20
Unwanted changes to this indicator would result in serious impacts (directly or indirectly) on ecological and human systems.	22	31	27	31	28	30
This indicator is important to me	24	31	25	34	31	28

**Total Score**	**150**	**175**	**151**	**207**	**180**	**168**
**Rank – short-listed?**	**6 – No**	**3 – Yes**	**5 – Yes**	**1 – Yes**	**2 – Yes**	**4 – Yes**

*Scores are the sum of individual participants’ scores for each criterion.

During discussions, Secchi depth was the one indicator workshop participants felt was sure to top the list as a key indicator. However, after assessing the indicators using the CBR process, results for the top five indicators were land use, wetland cover, carbon footprint, algae growth, and calcium; Secchi depth did not even make the list. This study confirmed that when a common set of criteria was used to assess indicators, a different set of indicators emerged than the set created without common principles to guide assessment.

### Discussion, recommendations and conclusions

To increase decisions informed by monitoring, the CBR process aims to align monitoring indicators with the priorities of decision-makers by encouraging diverse perspectives, participation and co-creation where possible. The list of VECs would ideally be co-created with representative community stakeholders. A ‘long list’ of monitoring indicators should be generated by diverse key stakeholders/subject matter experts and decision-makers (e.g., of differing political positions and scientific expertise).

Because the purpose of this study was to test the efficacy and impact of the CBR process, not to implement the process full-scale, criteria were provided to workshop participants rather than co-created with them. They were designed by the researcher to incorporate multiple needs (e.g., from ecological monitoring, social and policy perspectives). In future iterations these criteria will be designed by the stakeholders. Criteria were also weighted equally, since recent research concluded that applying weights to individual indicators did not significantly change the results of scoring [6]. However, since each context is different, future iterations should consult with key stakeholders as to whether unequal weighting is warranted.

A standard process of including or excluding indicators ensures degree of consistency despite regular fluctuations in capacity, thus improving the quality of monitoring. An unforeseen challenge at the workshop was achieving acknowledgement from participants that not all indicators were possible to address each year (as evidenced by different numbers of indicators having been reported year-to-year). Participants also discussed the challenge of balancing indicator quantity and quality. A concern was that the complexity of ecological systems would not be captured if indicators are oversimplified. Many participants agreed that maintaining a consistent set of indicators for ongoing monitoring would likely require reducing the current number of indicators.

In the same way indicators may need to be prioritized in times of fewer resources, monitoring practices may need to be assessed so that data collection sites are coordinated with prioritized indicators. For example, an indicator may not be ideal to measure the way it currently is (e.g., method and locations); however, another indicator studied at strategic locations may still provide desired insights. Alternatively, some monitoring programs use a consistent set of indicators but will do a rotation of sites across years. For example, in the Northumberland Strait estuaries (Gulf of Saint Lawrence in eastern Canada), eelgrass is measured in five estuaries per year on a five-year rotation so that the 25 estuaries are covered in a five-year period [7]. Considering changing indicators and sites together may improve cost-effectiveness and encourage the addressing of cumulative effects.

In addition, bias exists from individuals who are at the table to determine which indicators will be measured, but who do not represent all stakeholders in the watershed. The CBR indicator prioritization method is one way of many ways to address this bias. By ensuring indicator assessment criteria represent diverse perspectives, the representativeness of individuals assessing/selecting indicators is less important. This is not to say the criteria that were used were perfect. Rather, using broad (e.g., ecological, socio-political, economic) criteria agreed-upon by diverse watershed stakeholders is more likely to result in a list of indicators that responds to multiple needs and addresses multiple issues than without assessment criteria.

Like environmental assessment matrices, this process is transferrable to other contexts. The criteria used for scoring indicators, and whether weighting is applied (to criteria or components), can be determined for each context. Though this process was designed for ranking indicators, the same process can be applied for short-listing a lengthy list of VECs as well. Whether criteria should be standardized or specific to individual cases may also be explored. Of the criteria used in this study, the author suggests also incorporating ‘measures of success’ – for example, incorporating the criterion of ‘efficacy’, ‘reach’, or ‘influence’, evaluating whether the target audience (e.g., decision-makers) is reading or considering information reported from the monitoring program.

Having considered the above points, the CBR process for indicator selection was found to be an effective way to prioritize which monitoring indicators would be consistently used and which would be addressed as capacity permitted.

## Supplementary material and/or additional information

None.
